# Supplementary material for: PROTRIDER: protein abundance outlier detection from mass spectrometry-based proteomics data with a conditional autoencoder
Source: Bioinformatics. 2025 Nov 20;41(12):btaf628. doi: 10.1093/bioinformatics/btaf628 (PMC12931418; doi:10.1093/bioinformatics/btaf628)
Supplement: btaf628_Supplementary_Data [file btaf628_supplementary_data.zip › protrider_supp_material (1).pdf]

PROTRIDER: Protein abundance outlier detection from mass spectrometry-based proteomics data with a conditional autoencoder

Supplementary Figures

Daniela Klaproth-Andrade[1], Ines F. Scheller[1,2], Georgios Tsitsiridis[1], Stefan Loipfinger[1], Christian Mertes[1,3], Dmitrii Smirnov[2,3], Holger Prokisch[2,3,4], Vicente A. Yépez[1], Julien Gagneur[1,2,3@]

[1] TUM School of Computation, Information and Technology, Technical University of Munich, Garching, Germany

[2] Computational Health Center, Helmholtz Center Munich, Neuherberg, Germany

[3] Institute of Human Genetics, School of Medicine, Technical University of Munich, Munich, Germany

[4] German Center for Child and Adolescent Health (DZKJ), partner site Munich, Munich, Germany

[@] To whom correspondence should be addressed.

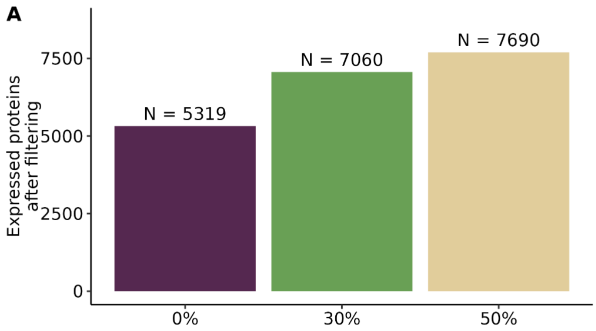

Fig. S1: Expressed proteins and rare variants per category on the mitochondrial disorder dataset. A) Number of expressed proteins after filtering out proteins with too many missing values per protein, for three cutoffs on the maximal allowed percentage of missing values: no missing values (0%, purple), at most 30% missing values (green), and at most 50% missing values per protein (beige).

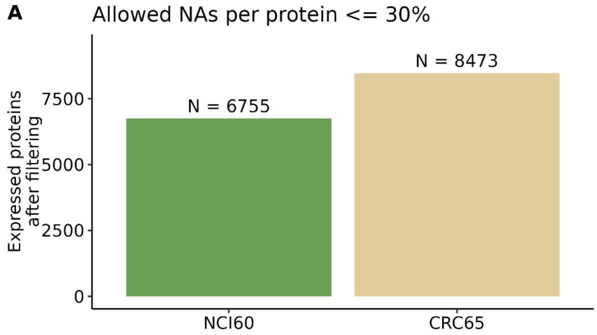

Fig. S2: Expressed proteins and rare variants per category on the tumor cell line datasets.
A) Number of expressed proteins after filtering out proteins with more than 30% missing values per protein, for the cell line panels NCI60 (green) and CRC65 (beige).

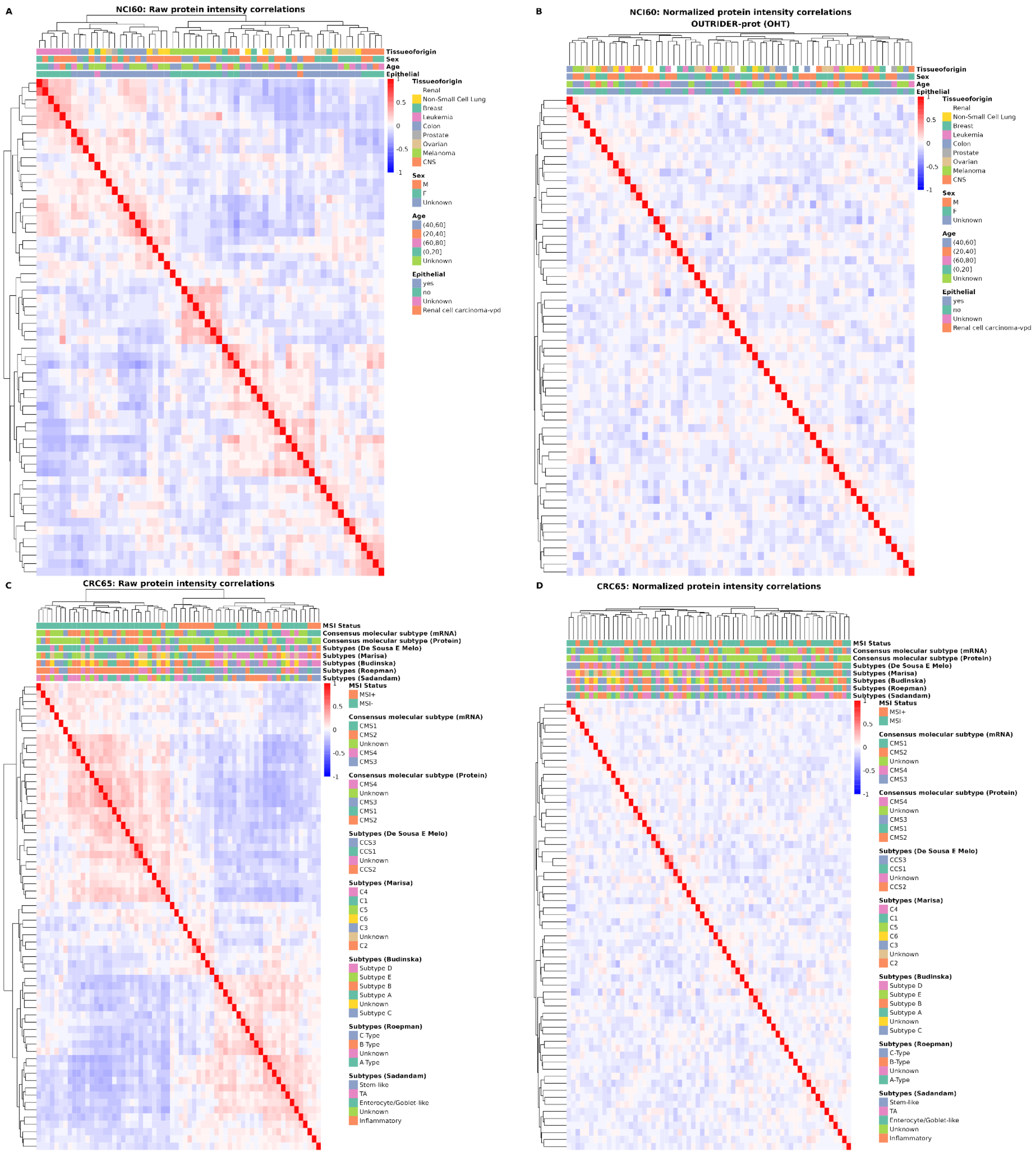

Fig. S3: Sample-sample correlations before and after autoencoder on the tumor cell line panels. A-B) Heatmaps of sample-sample correlations of protein log-transformed intensities before (A) and after the PROTRIDER autoencoder correction for the PROTRIDER version using OHT for finding the optimal encoding dimension (B) on the NCI60 cell line panel data. **C-D)** Same as (A) and (B), respectively, but for samples from the cell line panel CRC65. The within-batch pairwise sample correlation was reduced from 0.15 ± 0.11 (mean ± standard deviation) to 0.079 ± 0.062 for NCI60 and from 0.11 ± 0.074 to 0.05 ± 0.04 for CRC65'

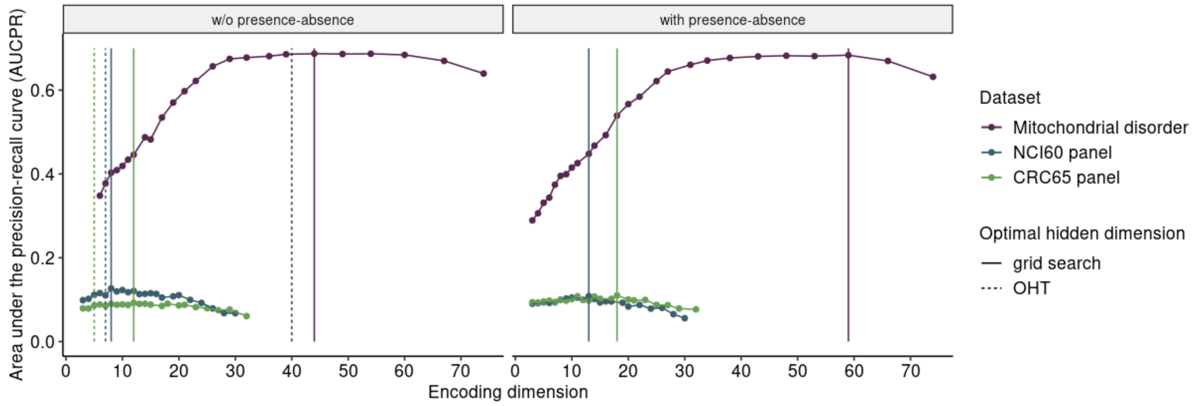

Fig. S4: Optimal encoding dimensions found by PROTRIDER and PROTRIDER (lite). The obtained area under the precision-recall curve of recovering injected outliers is plotted against the candidate encoding dimensions for the PROTRIDER approach without (left) and with (right) missingness modelling, i.e. presence-absence information. The vertical lines indicate the optimal encoding dimension found for each dataset with the grid search approach and the OHT approach.

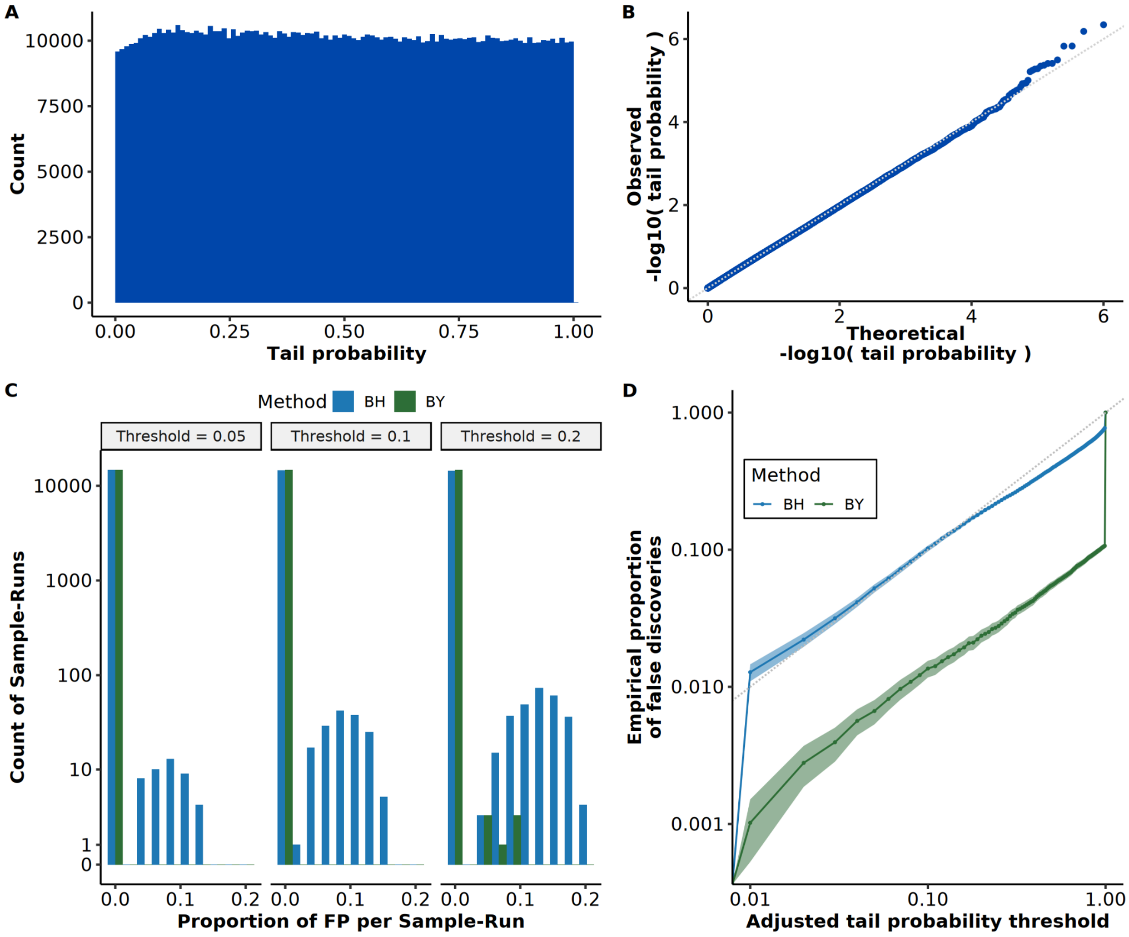

Fig. S5: Empirical assessment of proportions of false positive calls with PROTRIDER on simulated data without outliers. A) Histogram showing the distribution of two-sided tail probabilities computed by PROTRIDER fitted on a simulated intensity matrix under the assumption that model residuals follow a Student's t-distribution. **B)** Quantile-Quantile plot comparing the observed -log10(tail probabilities) to their theoretical quantiles under the null (uniform distribution). **C)** Histograms showing the proportions of false positives across simulated samples, stratified by significance threshold on adjusted tail probabilities with the method of Benhamini-Hochberg (BH, blue) and Benjamini-Yekutieli (BY, green). **D)** Adjusted tail probability thresholds (x-axis) against empirically observed proportions of false discoveries (y-axis) with the method of Benhamini-Hochberg (BH, blue) and Benjamini–Yekutieli (BY, green), estimated from 100 null simulations using residuals sampled from a fitted Student's t-distribution. The diagonal dashed line indicates perfect calibration. Ribbons mark 95% confidence intervals.

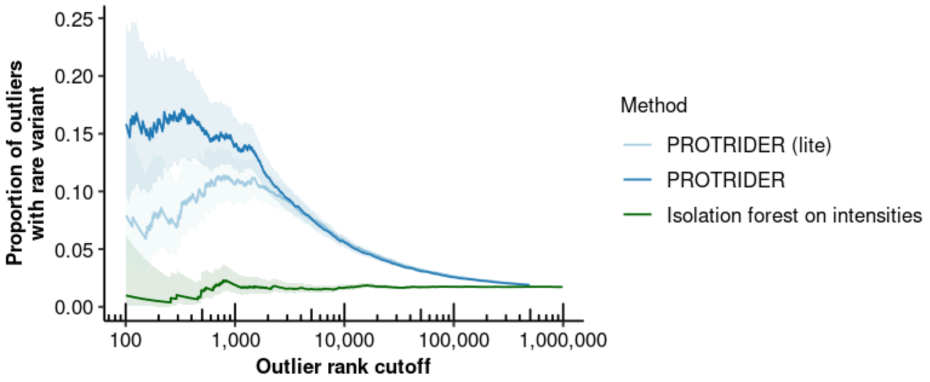

Fig. S6: PROTRIDER performance compared to an alternative approach based on Isolation forests. Proportion of outliers with at least one rare variant likely disrupting protein expression on the mitochondrial disorder dataset for underexpression outliers calls from PROTRIDER (blue), PROTRIDER (lite, light blue), and a protein-specific Isolation forest fitted on preprocessed intensities (dark green). Ribbons mark 95% confidence intervals.

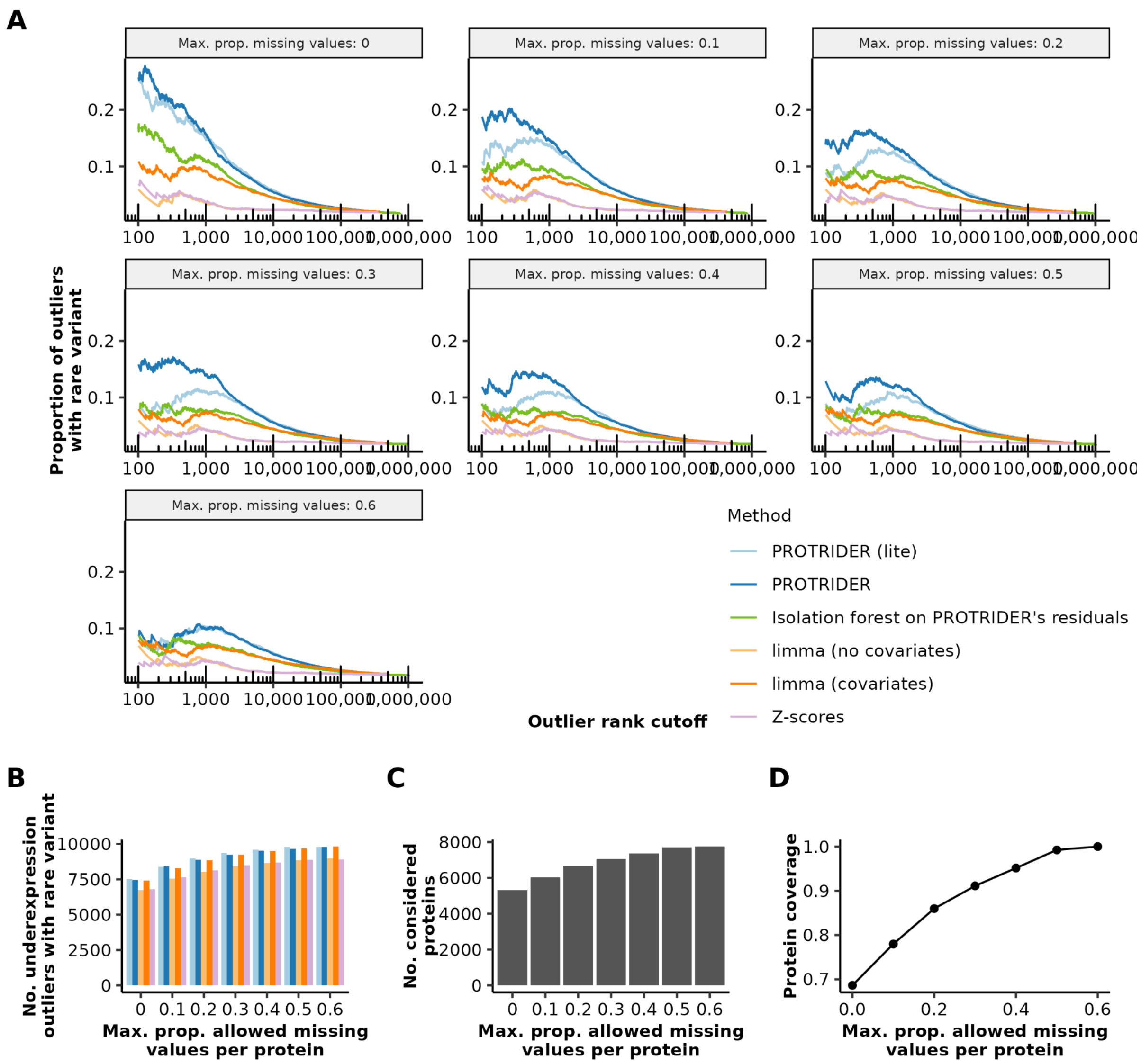

Fig. S7: PROTRIDER performance on the mitochondrial disorder dataset for different missing value thresholds. A, Proportion of outliers with at least one rare variant likely disrupting protein expression on the mitochondrial disorder dataset for underexpression outliers calls from PROTRIDER (blue), PROTRIDER (lite, light blue), the limma-based method with covariates (orange) and without covariates (light orange), and the Z-score-based method (violet) for different thresholds for removing proteins with more than the specified the maximal proportion of missing values per protein. Ribbons mark 95% confidence intervals. **B,** Total number of underexpression outliers with an adjusted tail probability of 0.1 or lower and with a rare variant likely disrupting protein expression for the different missing value thresholds. **C,** Total number of considered proteins for the different missing value thresholds. **D,** Protein coverage, i.e,. number of proteins considered over the total number of proteins in the mitochondrial disorder dataset, for the different missing value thresholds.

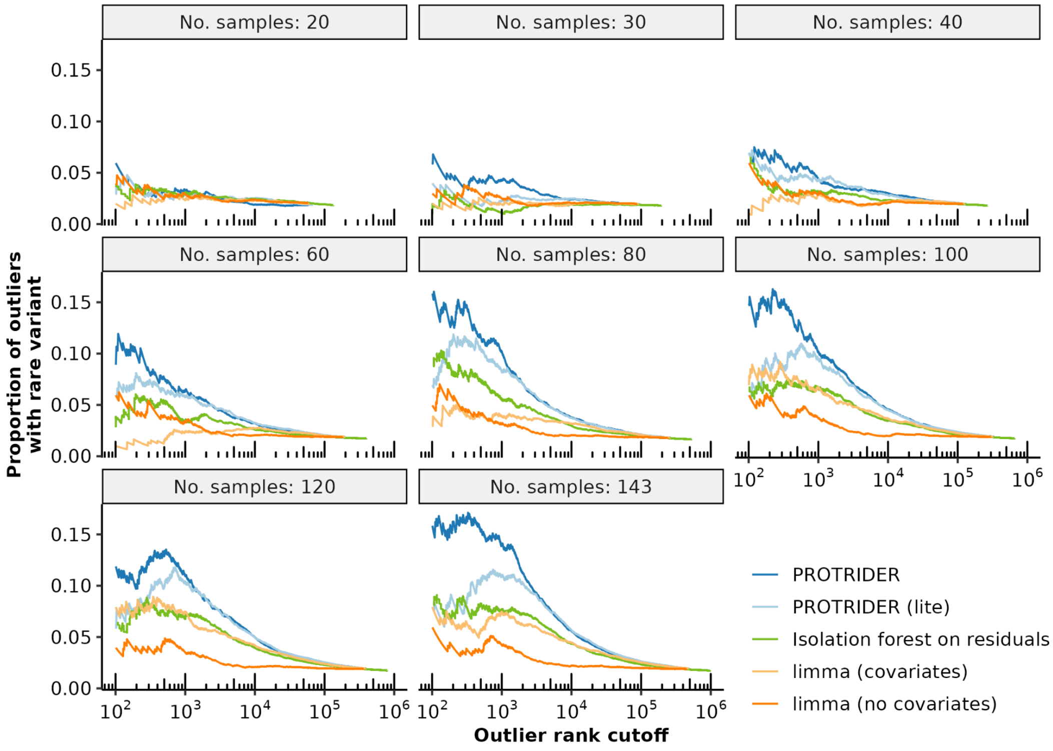

Fig. S8: Variant enrichment performance on random subsets of the mitochondrial disorder dataset. Proportion of outliers with at least one rare variant likely disrupting protein expression on the mitochondrial disorder dataset for underexpression outliers calls from PROTRIDER (blue), PROTRIDER (lite, light blue), the limma-based method with covariates (orange) and without covariates (light orange), and the isolation forest method on PROTRIDER's residuals method (light green), for different sample sizes obtained after randomly subsetting the mitochondrial disorder dataset.

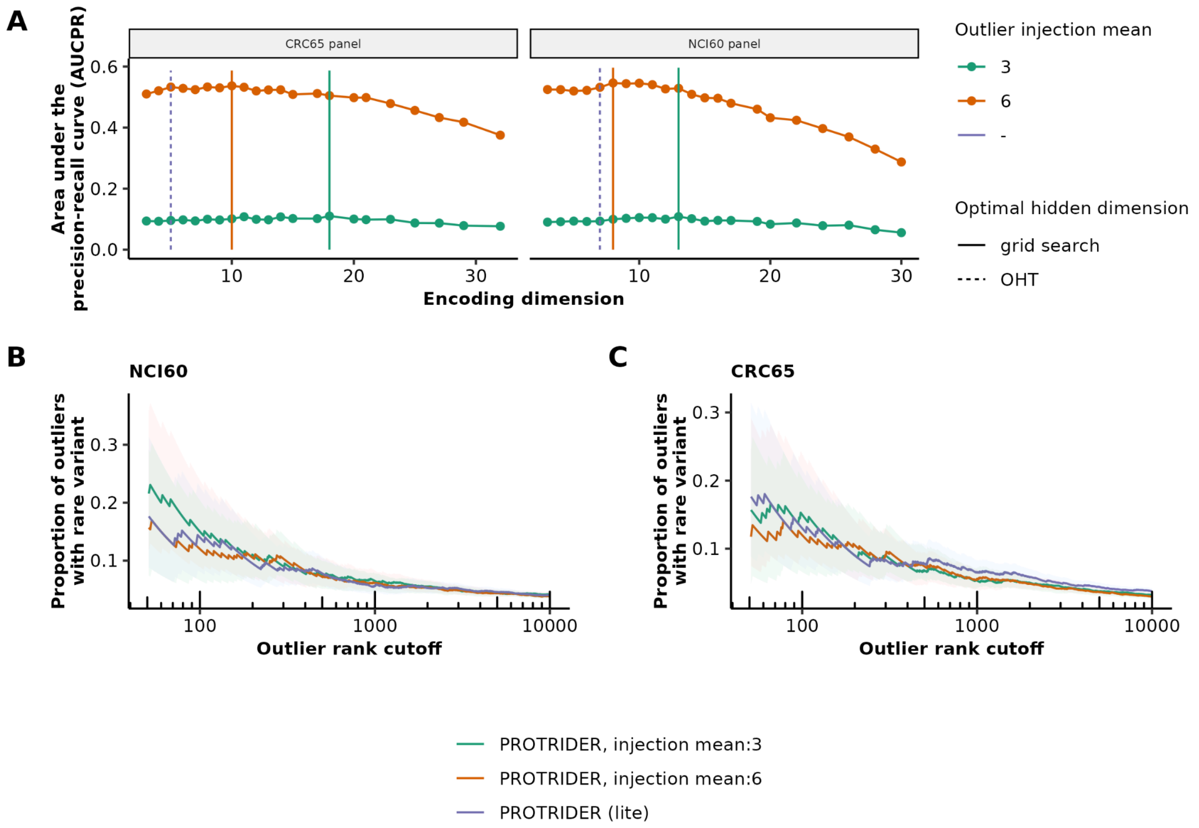

Fig. S9: PROTRIDER performance on the tumor cell line panels for different outlier injection settings. A, The obtained area under the precision-recall curve of recovering injected outliers with injection mean 3 (green) and 6 (orange) is plotted against the candidate encoding dimensions for PROTRIDER. The vertical lines indicate the optimal encoding dimension found for each dataset with the grid search approach and the OHT approach. **B,** Proportion of outliers with at least one rare variant likely disrupting protein expression on the mitochondrial disorder dataset for underexpression outlier calls from PROTRIDER based on an injection mean of 3 (green), 6 (orange), and PROTRIDER (lite, violet) for the tumor cell line panel NCI60. **C**, Same as for ,B but for the cell line panel CRC65

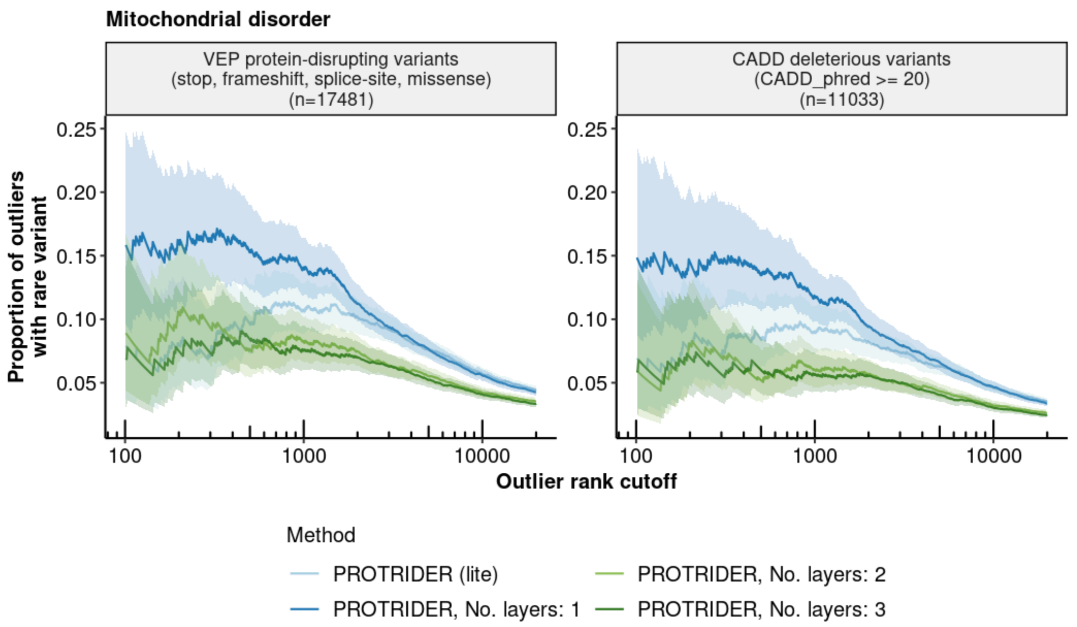

Fig. S10: Linear autoencoder model outperforms multi-layer autoencoder models. Proportion of outliers with at least one rare variant on the mitochondrial disorder dataset for underexpression outliers calls from PROTRIDER with different number of layers and, PROTRIDER (lite) with two sets of rare variant categories as ground truth proxies: i) VEP stop, frameshift, direct split-site, and missense variants and ii) CADD deleterious variants (PHRED score ≥ 20).

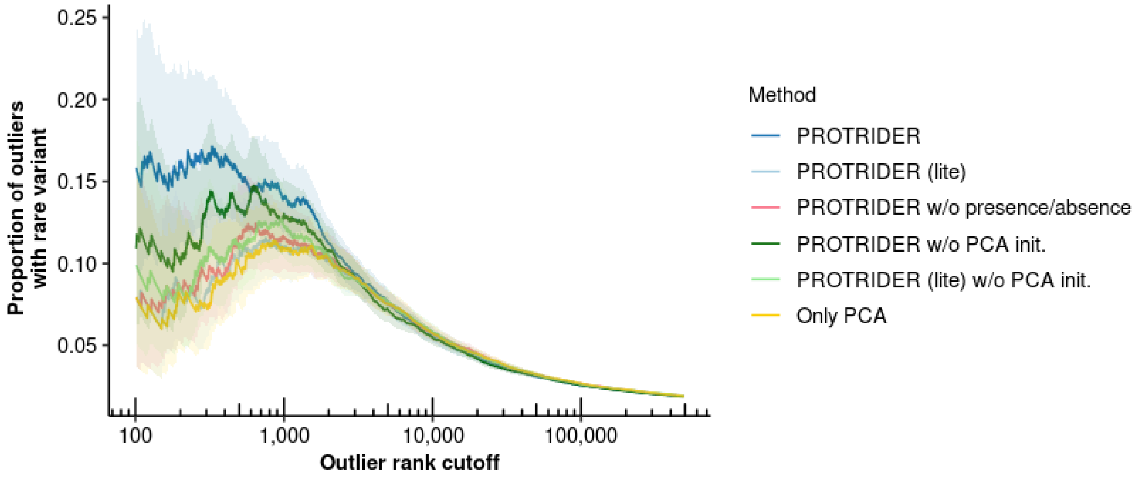

Fig. S11: PROTRIDER ablation study. Proportion of outliers with at least one rare VEP stop, frameshift, direct split-site, and missense variant on the mitochondrial disorder dataset for underexpression outlier calls from PROTRIDER (dark blue), PROTRIDER (lite, light blue), PROTRIDER without missingness modeling (rose), PROTRIDER without PCA initialization (light green and dark green), and only PCA projection with the number of principal components derived from the OHT procedure.

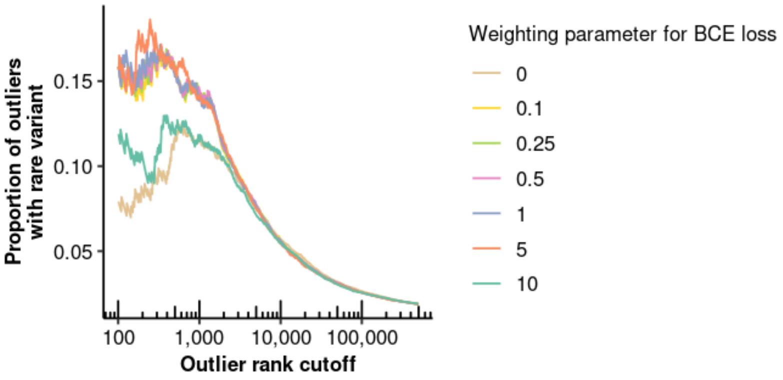

Fig. S12: Impact of weighting factor for binary cross entropy compared to regression loss. Proportion of outliers with at least one rare VEP stop, frameshift, direct split-site, and missense variant on the mitochondrial disorder dataset for underexpression outlier calls from PROTRIDER fitted with different weighting factors to aggregate the binary cross entropy and the mean squared error loss to optimize the prediction of protein intensities and presence probabilities. Each weighting factor is applied to the binary cross-entropy term and subsequently summed to the mean squared error term over observed protein intensities to compute the final loss term during model optimization (Methods).

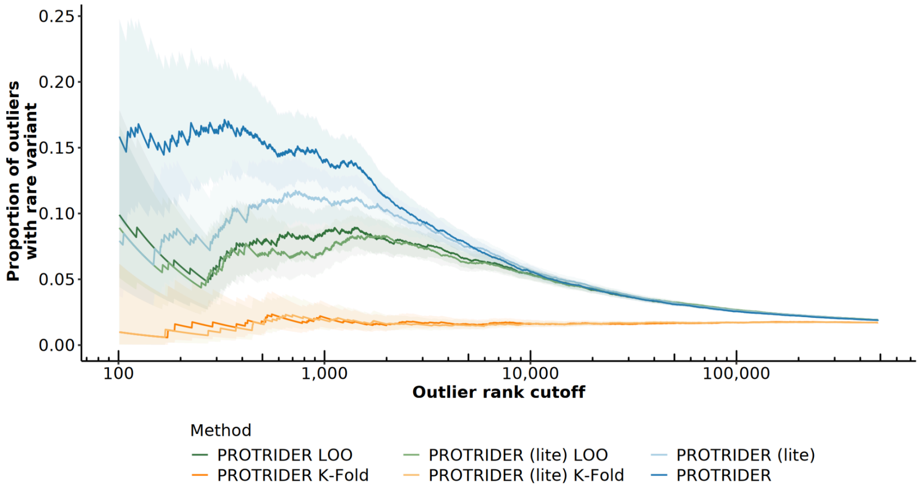

Fig. S13: Models trained on the full dataset perform best compared to cross-validation approaches. Proportion of underexpression outliers with at least one rare variant in the mitochondrial disorder dataset. Variants include VEP stop, frameshift, direct splice-site, and missense variants, used as ground truth proxies. The following models are compared: PROTRIDER (lite) with leave-one-out cross-validation (light green), *k*-fold cross-validation (light orange), and no cross-validation (light blue); PROTRIDER with leave-one-out cross-validation (green), *k*-fold cross-validation (orange), and no cross-validation (blue). Ribbons mark 95% confidence intervals.

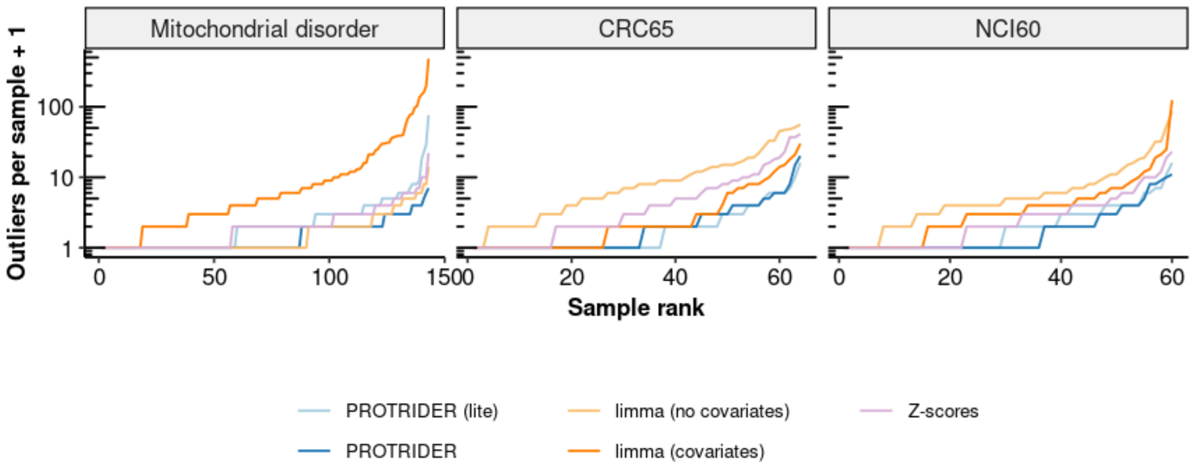

Fig. S14: Number of outliers per sample returned by PROTRIDER compared to limma-based approaches. Sorted number of protein abundance outliers per sample obtained by the methods: PROTRIDER (dark blue), PROTRIDER (lite, light blue), the limma-based approaches with (dark orange) and without covariates (light orange), and the Z-score-based (violet) approach on the three datasets (facets).

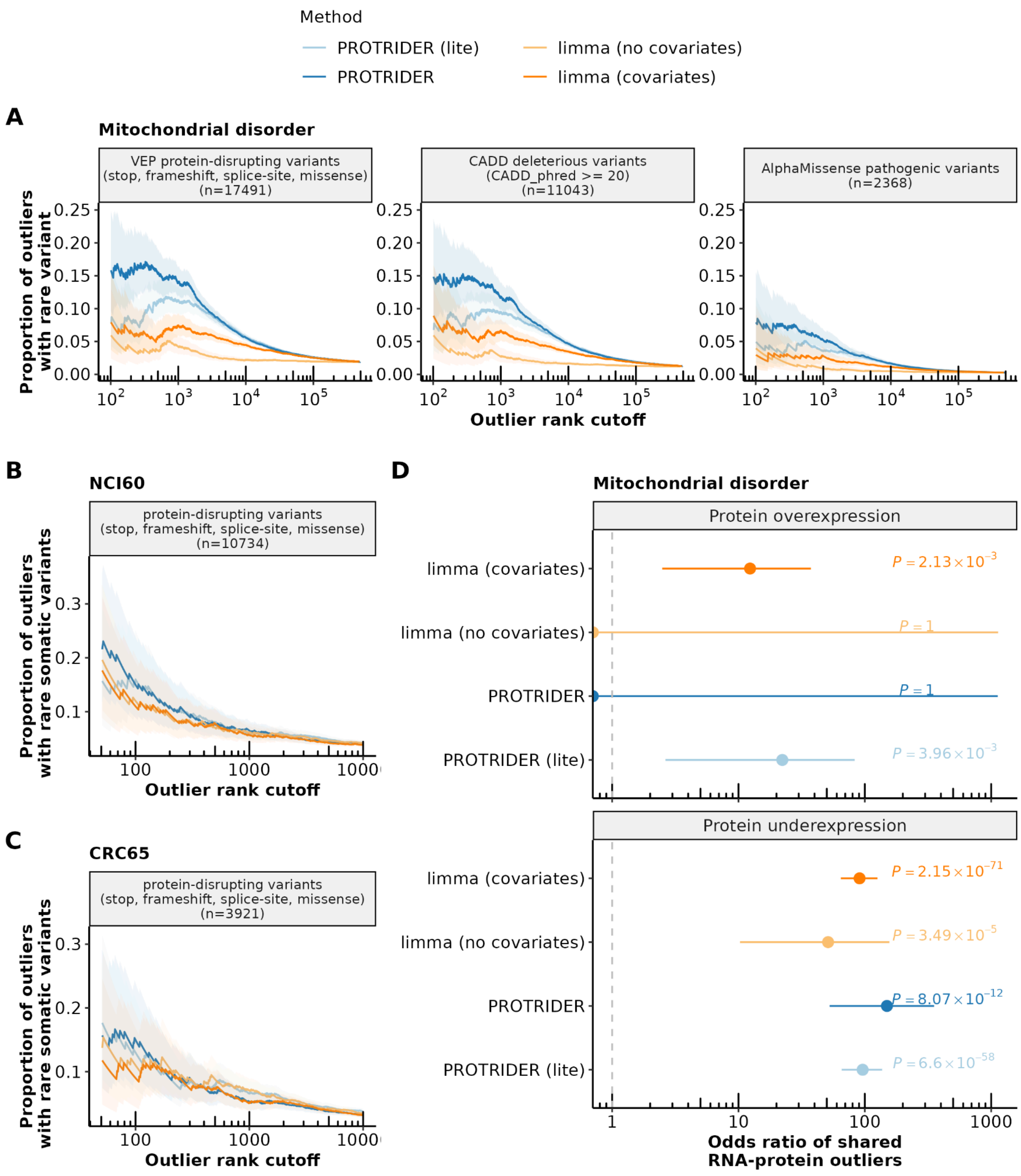

Fig. S15: PROTRIDER outperforms limma approaches on rare variant benchmarks. Same as Fig. 3, but for the comparison of PROTRIDER with limma with covariates (dark orange) and without covariates (light orange).

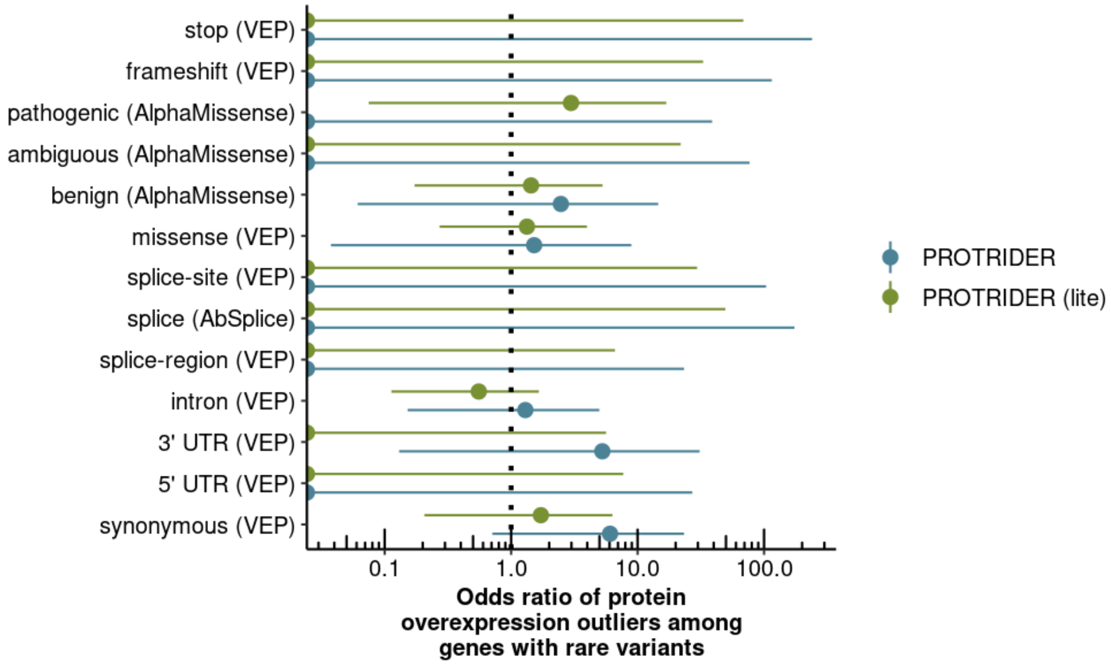

Fig. S16: Genetic determinants of protein overexpression outliers. Odds ratios and their 95% confidence intervals (Fisher's test) of the enrichment of the proportion for each variant category among overexpression outliers compared to the background proportion of the non-outliers.

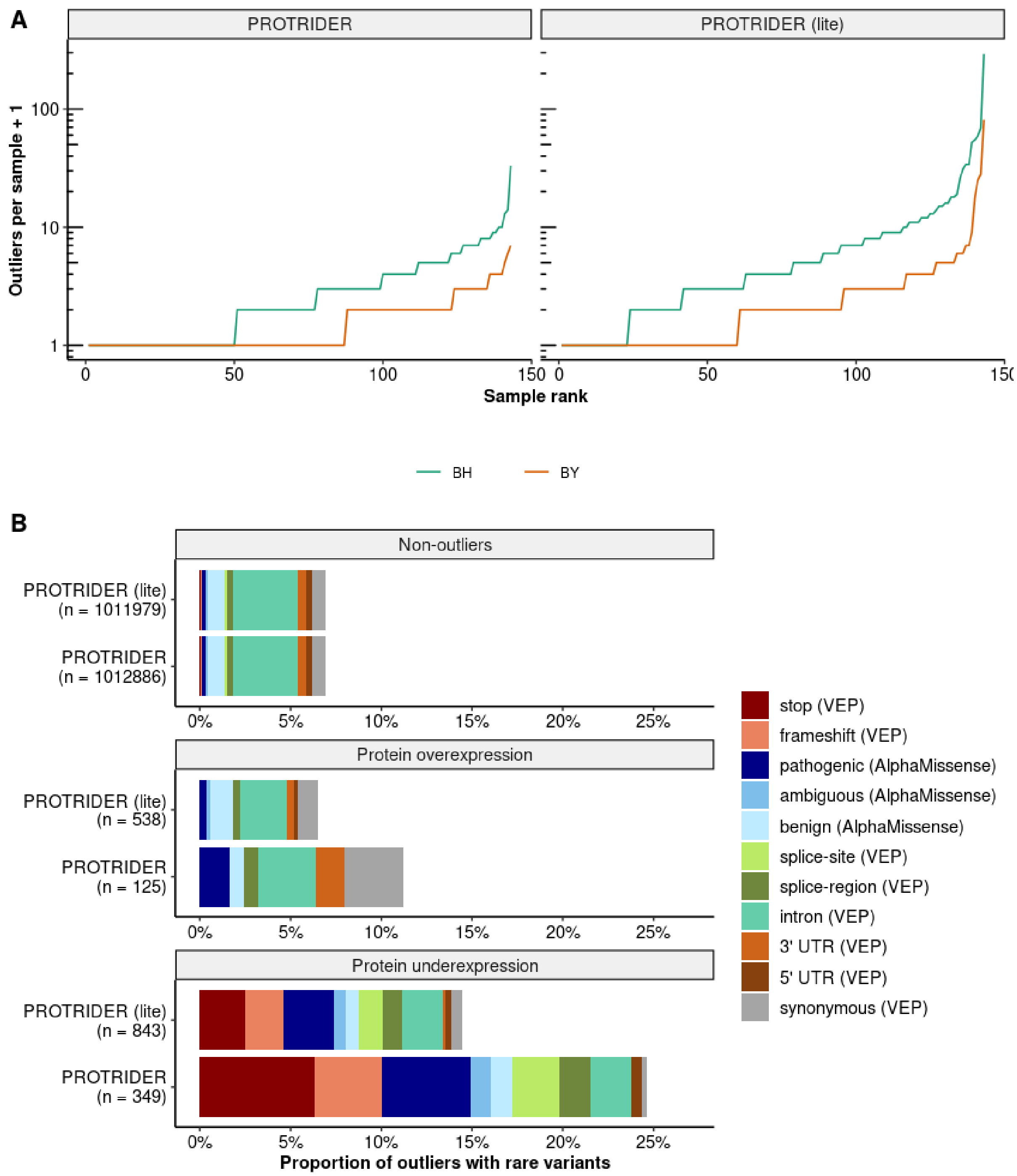

Fig. S17: Method comparison for tail probability adjustment. A, Sorted number of protein abundance outliers per sample obtained after adjusting the obtained tail probabilities with the methods of Benjamini and Yekutieli (BY) and Benjamini and Hochberg (BH) for the two PROTRIDER versions (facets). **B**, Same as Fig. 4A, but with the BH procedure for controlling the false discovery rate.
